# Clinical evaluation of high intensity interval training exercise in coronary heart disease patients after percutaneous coronary intervention: A protocol for systematic review and meta-analysis

**DOI:** 10.1097/MD.0000000000025472

**Published:** 2021-04-09

**Authors:** Xiaohui Cheng, Jian Huang, Jing Zhu, Hui Li, Haibo Gu

**Affiliations:** a Department of Nursing; b Department of Cardiology, The Second Affiliated Hospital of Soochow University, Jiangsu, China.

**Keywords:** coronary heart disease, high intensity interval training, meta-analysis, percutaneous coronary intervention, protocol, systematic review

## Abstract

**Background:**

Although the effect of high intensity interval training (HIIT) was gradually explicit, little is explored about the role and the validity of HIIT on patients following percutaneous coronary intervention (PCI). As far as we know, no evidence has been established to assess HIIT program for coronary heart disease (CHD) patients attending cardiac rehabilitation after PCI. Therefore, this systematic review and meta-analysis will be conducted to assess the efficacy and safety of HIIT program for CHD patients attending cardiac rehabilitation after PCI.

**Methods:**

Seven electronic databases including Web of Science, Embase, PubMed, Wanfang Data, Scopus, Science Direct, Cochrane Library will be searched in March 2021 by 2 independent reviewers. Study included in this systematic review and meta-analysis has to meet all of the following inclusion criteria in the PICOS order:

1. Participants: CHD patients after PCI;
2. Intervention: patients received HIIT;
3. Comparator: patients received other treatment;
4. Outcomes: cardiopulmonary function, lipid profiles and in- stent restenosis;
5. Study design: cohort trials.

Data extraction will be performed independently, and any conflict will be resolved before final analysis. Review Manager software (v 5.3; Cochrane Collaboration) is used for the meta-analysis.

**Results:**

The results of this research will be delivered in a peer-reviewed journal.

**Conclusion:**

This study expects to provide credible and scientific clinical evidence for the efficacy and safety of HIIT program for CHD patients attending cardiac rehabilitation after PCI.

**OSF registration number:**

10.17605/OSF.IO/AUMR6.

## 1 Introduction

For decades, percutaneous coronary intervention (PCI) for myocardial revascularization has been the standard treatment for coronary heart disease (CHD). Although PCI has been successful in alleviating epicardial coronary stenosis, microvascular dysfunction may prevent adequate coronary flow and myocardial perfusion, which may worsen clinical outcomes.^[1]^ There is a growing consensus that exercise has beneficial effects on patients with CHD, even those with severely impaired heart function, and that lack of exercise can accelerate the severity of heart failure.^[2]^ A recent meta-analysis has shown that cardiac rehabilitation programs can reduce overall mortality and cardiac mortality by 20% to 26% compared to standard medical services.^[3]^ Although exercise training has become a core element of cardiac rehabilitation programs, the amount, style, frequency, and intensity of exercise that will yield the best results for patients with heart disease remain controversial.^[4]^

High intensity interval training (HIIT) is one of the exercises recently explored in patients with low - to moderate-risk coronary heart disease. HIIT is a general term for time-efficient movement, with heart-based movement and multimodal movement intervals, and scattered active or passive recovery beats.^[5]^ The HIIT interval is typically between 85% and 100% of the maximum heart rate achieved during peak or symptomatic restricted movement testing. HIIT has been widely adopted by healthy populations as an alternative to moderate intensity continuous training; or the ubiquitous application of moderate intensity exercise utilized in rehabilitation settings.^[6,7]^ Several data indicate that HIIT is safe, well tolerated, effective, and particularly cost effective, so improving long-term compliance with cardiac rehabilitation programs may be a promising approach.^[7]^ In addition, previous data have shown that HIIT improves left ventricular compliance and contributes to an increase in systolic ejection and cardiac output compared to lower limb muscle training alone.^[8,9]^

Although the effect of HIIT was gradually explicit, little is explored about the role and the validity of HIIT on patients following PCI. As far as we know, no evidence has been established to assess HIIT program for CHD patients attending cardiac rehabilitation after PCI. Therefore, this systematic review and meta-analysis will be conducted to assess the efficacy and safety of HIIT program for CHD patients attending cardiac rehabilitation after PCI.

## 2 Materials and methods

### 2.1 Protocol registration

The prospective registration has been approved by the Open Science Framework registries (https://osf.io/aumr6), and the registration number is 10.17605/OSF.IO/AUMR6. The protocol is written following the Preferred Reporting Items for Systematic Reviews and Meta-Analyses Protocols (PRISMA-P) statement guidelines. No ethical approval is required in our study because all analyses will be based on aggregate data from previously published studies.

### 2.2 Search strategy

**Figure 1:**
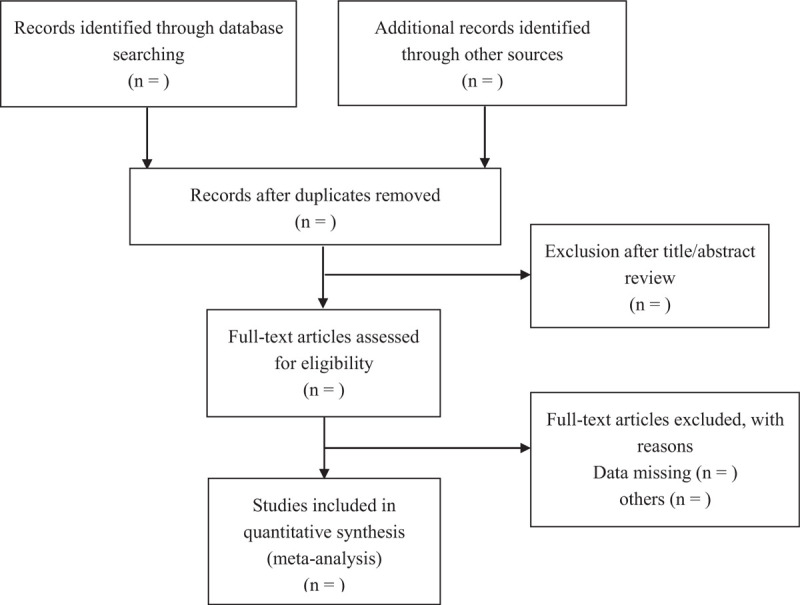
PRISMA flow diagram describing the selection process for relevant clinical trials used in this meta-analysis.

The following search terms will be used in Web of Science, Embase, PubMed, Wanfang Data, Scopus, Science Direct, Cochrane Library databases on March 2021, as the search algorithm: (coronary intervention) OR (coronary arteriography) OR (stent implantation) OR (stent placement) OR (percutaneous transluminal coronary angioplasty) AND (high-intensity interval training) OR (HIIT). The reference lists of the included studies will be also checked for additional studies that are not identified with the database search. There is no restriction in the dates of publication or language in the search (Fig. 1).

### 2.3 Inclusion and exclusion criteria

Study included in this systematic review and meta-analysis has to meet all of the following inclusion criteria in the PICOS order:

1. Participants: CHD patients after PCI;
2. Intervention: patients received HIIT;
3. Comparator: patients received other treatment;
4. Outcomes: cardiopulmonary function, lipid profiles and in- stent restenosis;
5. Study design: cohort trials.

The exclusion criteria were as follows:

1. studies which did not assessed the above outcomes;
2. no direct comparison of HIIT and other treatment;
3. studies with the following types: case reports, comments or letters, biochemical trials, protocols, conference abstracts, and reviews.

### 2.4 Data extraction

Data will be extracted by review of each study for population, mean age, gender, follow-up duration, study design, publishing date, intervention characteristics, and outcomes assessment. The 2 reviewers will create a study-specific speadsheet in Excel (Microsoft Corp.) for data collection. Data extraction will be performed independently, and any conflict will be resolved before final analysis. Any disagreements between the 2 reviewers will be discussed and, if necessary, the third author is referred to for arbitration. If the data are missing or can not be extracted directly, authors will be contacted by email. Otherwise, we calculate them with the guideline of Cochrane Handbook for Systematic Reviews of Interventions 5.1.0. If necessary, we will abandon the extraction of incomplete data.

### 2.5 Risk-of-bias assessment

Two independent reviewers will evaluate the risk of bias of the included randomized controlled trials on the basis of the guidelines of the Cochrane Handbook for Systematic Reviews of Interventions 5.1.0 by using Cochrane Collaboration tool for assessing the risk of bias. The score consists of 7 items, including random sequence generation, allocation concealment, blinding of participants and personnel, blinding of outcome assessment, incomplete outcome data, selective reporting, and other bias. When evaluating the methodological quality of retrospective studies, the Methodological Index for Non-randomized Studies (MINORS) criteria will be used.

### 2.6 Statistical analysis

Review Manager software (v 5.3; Cochrane Collaboration) is used for the meta-analysis. Extracted data are entered into Review Manager by the first independent author and checked by the second independent author. Risk ratio with a 95% confidence interval or standardized mean difference with 95% CI are assessed for dichotomous outcomes or continuous outcomes, respectively. The heterogeneity is assessed by using the *Q* test and *I*^2^ statistic. An *I*^2^ value of <25% is chosen to represent low heterogeneity and an *I*^2^ value of >75% to indicate high heterogeneity. All outcomes are pooled on random-effect model. A *P* value of <.05 is considered to be statistically significant.

## 3 Discussion

Although the effect of HIIT was gradually explicit, little is explored about the role and the validity of HIIT on patients following PCI. As far as we know, no evidence has been established to assess HIIT program for CHD patients attending cardiac rehabilitation after PCI. Therefore, this systematic review and meta-analysis will be conducted to assess the efficacy and safety of HIIT program for CHD patients attending cardiac rehabilitation after PCI. We will conduct this systematic review and meta-analysis according to the PRISMA guidelines. Two independent authors will use a highly sensitive search strategy to identify the trials in the 7 main databases and supplemented it by manually searching for studies related to the topic and the reference list of included studies. There is no restriction in the dates of publication or language in the search for the current review, and thus publication and language bias can be minimized. In accordance with recommendations of GRADE, the quality of the evidence is carefully evaluated in this review, and thus generating a precise level of confidence of our results. This study expects to provide credible and scientific clinical evidence for the efficacy and safety of HIIT program for CHD patients attending cardiac rehabilitation after PCI.

## Author contributions

**Conceptualization:** Jing Zhu, Jian Huang.

**Data curation:** Xiaohui Cheng, Jian Huang.

**Formal analysis:** Xiaohui Cheng, Jian Huang.

**Funding acquisition:** Haibo Gu.

**Investigation:** Xiaohui Cheng, Jian Huang, Hui Li.

**Methodology:** Jian Huang, Jing Zhu.

**Project administration:** Jing Zhu, Hui Li, Haibo Gu.

**Resources:** Hui Li, Haibo Gu.

**Software:** Jing Zhu, Hui Li.

**Supervision:** Haibo Gu.

**Validation:** Jian Huang.

**Visualization:** Xiaohui Cheng.

**Writing – original draft:** Xiaohui Cheng, Jian Huang.

**Writing – review & editing:** Haibo Gu.
